# Cerebrospinal Fluid β-Amyloid_1–42_ Levels in the Differential Diagnosis of Alzheimer’s Disease—Systematic Review and Meta-Analysis

**DOI:** 10.1371/journal.pone.0116802

**Published:** 2015-02-24

**Authors:** Jin-A Mo, Ju-Hee Lim, Ah-Ram Sul, Min Lee, Young Chul Youn, Hee-Jin Kim

**Affiliations:** 1 National Evidence-Based Health Care Collaborating Agency, Seoul, Korea; 2 Department of Nursing, Inha University College of Medicine, Incheon, Korea; 3 Department of Neurology, Chung-Ang University College of Medicine, Seoul, Korea; 4 Department of Neurology, Hanyang University College of Medicine, Seoul, Korea; Fatebenefratelli Foundation for Health Research and Education, ITALY

## Abstract

**Objectives:**

The purpose of this study was to carry out systematic review of the literature and meta-analysis to evaluate the diagnostic utility of cerebrospinal fluid (CSF) levels of the 42 amino acid form of amyloid-beta (Aβ_1–42_) as a biomarker for differentiating Alzheimer’s disease (AD) from non-AD dementia.

**Methods:**

**Design.** Systematic literature review was used to evaluate the effectiveness of the Aβ for the diagnosis of AD. The Scottish Intercollegiate Guidelines Network (SIGN) tool was used to evaluate independently the quality of the studies.

**Data sources.** The literature review covered from January 1, 2004, to October 22, 2013, and searched eight domestic databases including Korea Med and international databases including Ovid-MEDLINE, EMBASE, and Cochrane Library.

**Data Extraction and Synthesis.** Primary criteria for inclusion were valid studies on (i) patients with mild cognitive impairment with confirmed or suspected AD and non-AD dementia, and (ii) assessment of Aβ_1–42_ levels using appropriate comparative tests.

**Results:**

A total of 17 diagnostic evaluation studies were identified in which levels of CSF Aβ_1–42_ were assessed. Meta-analysis was performed on 11 robust studies that compared confirmed AD (*n* = 2211) with healthy individuals (*n* = 1030), 10 studies that compared AD with non-AD dementias (*n* = 627), and 5 studies that compared amnestic mild cognitive impairment (*n* = 1133) with non-amnestic type subjects (*n* = 1276). Overall, the CSF Aβ_1–42_ levels were reduced in AD compared to controls or non-AD dementia. The effectiveness of test was evaluated for diagnostic accuracy (pooled sensitivity, 0.80 (95% CI 0.78–0.82); pooled specificity, 0.76 (95% CI 0.74–0.78).

**Conclusions:**

Reduced CSF Aβ_1–42_ levels are of potential utility in the differential diagnosis of AD versus non-AD dementias and controls. Diagnostic accuracy was high in AD versus healthy controls. However, differential diagnosis for MCI or non-AD might be evaluated by other biomarkers.

## Introduction

A substantial proportion of current therapeutic development in AD focuses on therapies targeting the Aβ peptide or Aβ aggregates, the core pathology of AD [1,2]. However, large-scale clinical trials of Aβ removal by immunological or pharmacologic means have yielded no reproducible benefits [2]. There are two routes to resolve this dilemma. First, anti-Aβ therapies (and perhaps anti-tau therapies) might be conducted on minimally affected individuals (secondary prevention in stages 1/2). A second strategy is to develop therapies that are likely to be of benefit in symptomatic patients (i.e., in a preclinical stage 3 or prodromal AD) [2]. Therefore, further development of AD therapeutics will require the establishment of biomarkers that accurately reflect the progression of AD pathology, thereby permitting early diagnosis of AD and facilitating drug trials selectively targeting the early predementia stages of the disease [3].

The sampling of cerebrospinal fluid (CSF) represents the most direct and convenient methods to study the biochemical changes occurring in the central nervous system. Aβ_1–42_, tau, and phosphorylated forms of tau have emerged as attractive diagnostic and prognostic CSF biomarkers for ongoing AD research [4,5]. Decreased CSF Aβ_1–42_ has been proposed as an useful diagnostic tool for AD [4]. It has been reported that the mean level of Aβ_1–42_ in the CSF are reduced to around 50% in subjects with AD relative to age-matched controls against initial prediction [4], and diagnosis of AD has evolved towards separate categories of preclinical and overt dementia based on levels of CSF Aβ_1–42_ [6]. However, CSF Aβ_1–42_ levels have been reported to fluctuate over time in a cohort of old and young individuals [7], and no absolute threshold has been identified that would differentiate between mild cognitive impairment and AD in mildly symptomatic individuals [8].

In the present study we aimed to review systematically the reported association between CSF Aβ_1–42_ and AD with a view to evaluating the clinical usefulness of CSF Aβ_1–42_ in the differential diagnosis of AD versus non-AD cognitive impairment.

## Methods

Systematic literature review was performed according to the reporting guidelines of the Arbitration Act Handbook (Hoggins and Green) as proposed by the Cochrane Union (Cochrane collaboration) and the PRISMA (Preferred Reporting Items for Systematic Reviews and Meta-Analyses) group [9]. In this study all researchers were recommended by the Korean Medical Association: these comprised a specialist of the Korean Ministry of Health and Welfare, two experts in laboratory medicine, two neurologists, and one neurological surgeon. Six meetings of all experts were held (three times in writing, three times in person) to (i) establish selection criteria, (ii) review studies selected for inclusion, (iii) overview data extraction, (iv) refine and validate the conclusions of the study.

### 1. S*ystematic literature review*

Systematic literature searching was performed in the Ovid-MEDLINE, EMBASE, and Cochrane Library data bases, as well as Korea Med, and was completed on October 22, 2013. Medline searching was conducted to locate all studies published in English and Korean from January 2004 to March 2013 using MeSH terms ‘Alzheimer disease/diagnosis’ [Mesh] AND ‘sensitivity and specificity’ [Mesh] AND (imaging OR biomarkers) and (‘dementia/diagnosis’ [Mesh] AND ‘biological markers/cerebrospinal fluid’ [Mesh]) OR ‘AD/diagnosis’ [Mesh]) AND ‘([beta or amyloid] adj2 42). mp.OR (amyloid adj2 [beta or 42]).mp.)’ in Ovid-EMBASE (S1 Table). All 369 abstracts were reviewed using a combination of the search terms. The Patients—Intervention—Comparators—Outcomes (PICO) and search strategy was drafted. Study groups included patients with suspected mild cognitive impairment and/or AD, and study selection focused on reports that included measurements of Aβ levels. The reference standard was clinical diagnosis with medical results being followed up for more than 1 year. Literature searches using MEDLINE and EMBASE are summarized in S1 Table. One report (Swedish Council on Technology Assessment 2008) was identified by searching the Cochrane Library and other databases for ‘Aβ_1–42_’.

### 2. Inclusion and exclusion criteria for selected documents

1. Inclusion criteria
  - Research on mild cognitive impairment (MCI) or patients with suspected or confirmed AD
  - National Institute of Neurological and Communicative Diseases and Stroke/Alzheimer’s Disease and Related Disorders Association (NINCDS-ADRDA) criteria [10] and Consortium to Establish a Registry for Alzheimer’s Disease (CERAD) [11] for AD
  - Diagnostic and Statistical Manual of Mental Disorders, fourth edition (DSM-IV)[12]for MCI and Other dementia
  - Studies using Aβ_1–42_ testing
  - Comparative research using appropriate tests
  - For predictive accuracy of reporting, studies with more than 1 year follow-up
  - Research paper using appropriate inspection techniques (eg, diagnostic tools as ELISA immunoassay, amyloid PET, biopsy or autopsy)
  - Research paper since 2004
2. Exclusion criteria
  - Reports restricted to treatment or preclinical animal studies
  - Unpublished studies
  - Non-research articles (non-systematic reviews, editorials, letters, comments, opinion pieces, congress or conference material, guidelines, notes, news articles, abstracts)
  - Studies published only as abstracts or case reports

**Fig 1:**
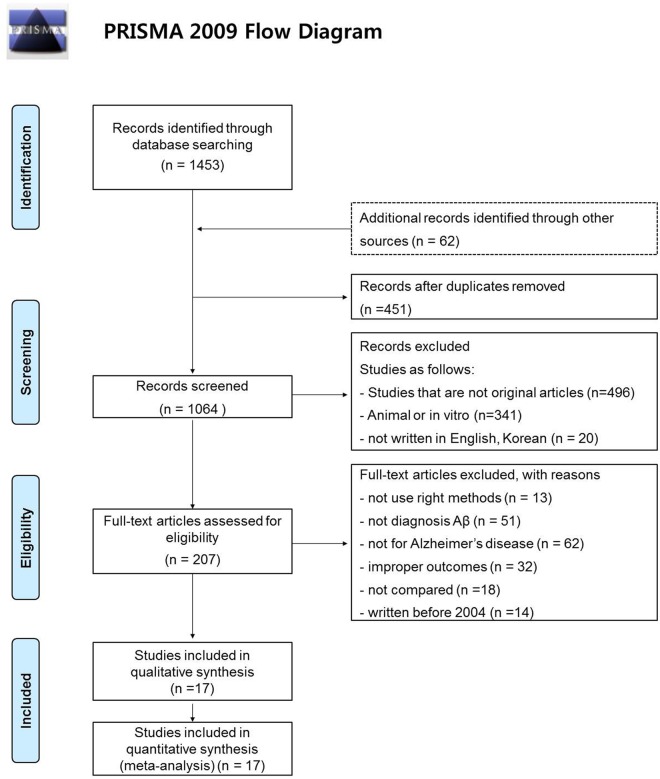
Literature search algorithm. Searching through the literature identified 1515 documents; a further 62 documents were identified using hand searching. Of these, 1097 documents met our exclusion criteria. 451documents duplicated data from other reports and were also excluded. A total of 17studieswere included in the final evaluation.

Searching through the literature identified 1515 documents; a further 62 documents were identified using hand searching. Among these, 1097 documents met our exclusion criteria. 451duplicated data from other reports were also excluded. A total of 17studies were included in the final evaluation (Fig. 1).

### 3. Quality of documents

The quality assessment tool selected for literature selection was adopted from the UK Scottish Intercollegiate Guidelines (SIGN) ‘Methodology Checklist’ (2004 March). SIGN is a systematic evaluation tool for the quality of the original literature and divides reports into randomized controlled trials, cohort studies, case—control studies, diagnostic assessments, and economic evaluation studies. Most of the literature on health technology assessment comprises non-randomized clinical trials and observational studies, and selection criteria were adapted accordingly (Table 1). Each stage of categorization was performed independently by two evaluators; their joint recommendations graded reports as summarized in Table 2. The present study excluded ‘The Swedish Council on Technology Assessment in Health Care Study’ in view of limitations as follows: (i) the study did not fulfil PICO standards; (ii) database searching was based on the references of pre-selected literature; (iii) the study included diverse controls ranging from non-AD dementias to other psychiatric or neurological disorders.

**Table 1:** Levels of Evidence (SIGN 50).

1++	High-quality meta-analyses, systematic reviews of RCTs, or RCTs with a very low risk of bias
1+	Well-conducted meta-analyses, systematic reviews, or RCTs with a low risk of bias
1-	Meta-analyses, systematic reviews, or RCTs with a high risk of bias
2++	High-quality systematic reviews of case—control or cohort studies High-quality case—control or cohort studies with a very low risk of confounding or bias and a high probability that the relationship is causal
2+	Well-conducted case—control or cohort studies with a low risk of confounding or bias and a moderate probability that the relationship is causal
2-	Case—control or cohort studies with a high risk of confounding or bias and a significant risk that the relationship is not causal
3	Non-analytic studies, e.g., case reports, case series
4	Expert opinion

Abbreviation: RCT, randomized controlled trial.

**Table 2:** Grades of Recommendations (Health Insurance Review Agency 2005)[15].

A	At least one meta-analysis, systematic review, or RCT rated as 1++, and directly applicable to the target population; or a body of evidence consisting principally of studies rated as 1+, directly applicable to the target population, and demonstrating overall consistency of results
B	A body of evidence including studies rated as 2++, directly applicable to the target population, and demonstrating overall consistency of results; or extrapolated evidence from studies rated as 1++ or 1+
C	A body of evidence including studies rated as 2+, directly applicable to the target population and demonstrating overall consistency of results; or extrapolated evidence from studies rated as 2++
D	Evidence level 3 or 4; or extrapolated evidence from studies rated as 2+

### 4. Data Extraction

Because documents put forward for evaluation comprised more than one type of study, data extraction was repeated several times and analyzed by two evaluators. Selection and categorization were performed in consultation with other researchers who advised on problem resolution. The data were then categorized according to type of data, study characteristics, and the reliability of the techniques employed. Final extraction of data from validated primary sources was performed by two evaluators.

### 5. Statistical Analyses

Funnel plot was used to address publication bias. Sensitivity testing was also conducted to assess the magnitude of publication bias, which was determined using a fail-safe number, defined as the minimum number of patients with non- significant findings that are needed to overturn the conclusion of a meta-analysis [13–15]. Larger fail-safe numbers indicate that the results are less prone to publication bias. For each outcome we tested the heterogeneity of results across the studies using “I^2^”. If significant heterogeneity was observed (p<.10), a random effects model-which assigns a weight to each study based on individual study variance as well as between study variance- was used to pool the results together. Also Mann-Whitney test was used to compare numerical values of β-amyloid levels between different reports in same disease categories (χ^2^). Confidence intervals were determined using the means and standard deviations reported in each document. Meta-analysis was performed to assess the overall diagnostic accuracy of the pooled reports based on the random effects model. In addition, the fail-safe Number was calculated manually with EXCEL, suggested by Corwin [16]. SPSS (Statistical Package for the Social Sciences) 21.0 (SPSS/IBM Inc, New York) was used to recalculate the reported the χ^2^ values. Revman 5.0 Meta DiSc 1.4 version (Hospital Universtario Ramony Cajal, Madrid, Spain) was subsequently used for meta-analysis of the entire dataset.

## Results

Following systematic analysis of the literature and retrieval of primary data, meta-analysis was performed on eleven robust studies that compared Aβ_1–42_ levels in AD (*n* = 2211) with healthy individuals (*n* = 1030), 10 studies that compared AD with non-AD dementias (*n* = 627), and five studies that compared a-MCI (amnestic mild cognitive impairment) (*n* = 1133) with na-MCI (non-amnestic mild cognitive impairment) subjects (*n* = 1276). The present evaluation is therefore based on the results of 17 published studies (Fig. 1). The primary documents and the extracted data are listed in Table 3. All selected paper used ELISA Kit of *Innotest* kind as a test tools, despite not limited to scan tool and the type of the selected documents. Range of test was 125 ~ 2000pg/mL, respectively and threshold was varied from 290 to 679pg/mL according to each document.

**Table 3:** Selected Documents Reporting CSF Aβ_1–42_ Measurements in AD and MCI.

First author	Publication year	Patients	Aβ_1–42_		N	Age	MMSE	Cutting point	TP	FP	FN	TN		Level of evidence
			Mean	SD										
Vos [34]	2013	a-MCI	550	267	399	70.7±7.8	26.5±2.5	500	-	-	-	-		2++
		na-MCI	624	283	226	70.7±7.6	27.5±2.1		-	-	-	-		
Dumurgier [35]	2013	AD	426.8	119.5	515	71.5±9.5	18.8±6.2		Reference					2++
		Other	605.9	260.6	365	66.7±11.4	21.6±0.0	515	99 (52.1)	19 (10.0)	37 (19.5)	35 (18.4)	Paris	
								368	207 (49.3)	38 (9.0)	85 (20.2)	90 (21.5)	Lilly	
								582	115 (33.8)	68 (20.0)	42 (12.4)	115 (33.8)	Mong	
Park [36]	2013	AD	194	88.7	17	59.0±8.0	15.0±7.0	290	Reference					2++
		Other	184.5	121	9	70.0±9.0	18.0±8.0		-	-	-	-		
		Control	383.5	101.8	12	63.0±9.0	28.0±1.0		15 (51.7)	2 (6.9)	2 (6.9)	10 (34.5)		
Reijn [37]	2007	AD	401	74	69	69.0±0.0	20.5±0.0	67	Reference					2++
		Other	570	238.5	26	69.5±0.0	21.5±0.0		60 (63.2)	10 (10.5)	9 (9.5)	16 (16.8)		
		Control	810	170	55	59.0±0.0	-		59 (47.6)	9 (7.3)	10 (8.1)	46 (37.0)		
Lewczuk [38]	2004	AD	370.5	75.5	22	68.0±0.0	14.0±0.0	550	Reference					2++
		Other	650	357.5	11	75.0±0.0	22.0±0.0		19 (57.6)	2 (6.1)	3 (11.0)	9 (25.3)		
		Control	865	256	35	61.0±0.0	-		22 (38.6)	6 (10.5)	0 (0)	29 (50.9)		
Schoonenboom [39]	2004	AD	307	200.5	47	59.0±0.0	20.0±0.0	413	Reference					2++
		Other	603	413.5	28	60.0±0.0	25.0±0.0		40 (53.3)	7 (9.3)	7 (9.3)	21 (28.1)		
		Control	604	443.5	21	62.0±0.0	29.0±0.0		40 (58.8)	1 (1.5)	7 (10.3)	20 (29.4)		
Le Bastard [40]	2013	AD	355	353	51	75.0±13.0	11.0±7.0	539	Reference					2+
		Other	610	406	95	72.0±10.0	10.0±9.0		43 (29.5)	26 (17.8)	8 (5.5)	69 (47.2)		
		Control	699	417	95	47.0±17.0	-		48 (32.8)	11 (7.5)	3 (2.1)	84 (57.6)		
Buchhave [41]	2009	AD	296	211.5	529	74.0±7.2	20.4±5.6	-	-	-	-	-		2+
		Control	651	168	34	72.0±8.3	28.7±1.2		-	-	-	-		
Mattsson [42]	2009	AD	370	211.5	529	71.0±0.0	22.0±0.0	482	Reference					2+
		a-MCI	356	163.1	271	72.0±0.0	27.0±0.0		223 (29.8)	134 (17.9)	47 (6.3)	345 (46.0)		
		na-MCI	579	216.5	479	68.0±0.0	27.0±0.0		-	-	-	-		
		Control	675	285.8	304	67.0±0.0	29.0±0.0		-	-	-	-		
Smach [43]	2009	AD	400	370	73	73.0±0.0	14.0±0.0	505	Reference					2+
		Other	680	315	35	69.0±0.0	18.0±0.0		60 (55.5)	10 (93)	13 (12.0)	25 (23.2)		
		Control	1020	230	38	72.0±0.0	28.0±0.0		60 (54.1)	3 (2.7)	13 (11.7)	35 (31.5)		
Herukka [44]	2008	a-MCI	392	154	13	-	-	450	Reference					2+
		na-MCI	670	249	8	-	-		6 (28.6)	2 (9.5)	2 (9.5)	11 (52.4)		
Kapaki [45]	2007	AD	422	149	67	66.0±10.0	18.0±0.0	61	Reference					2+
		Other	400	219	18	69.0±14.0	21.0±0.0		61 (71.8)	10 (11.8)	6 (0.7)	8 (15.7)		
		Control	721	228	72	64.0±11.0	29.0±0.0		48 (35.3)	8 (5.9)	19 (13.7)	61 (45.1)		
Kapaki [46]	2005	AD	387	77	33	63.0±11.0	23.0±0.0	562	Reference					2+
		Other	800	174	20	60.0±12.0	25.0±0.0		28 (52.8)	4 (7.5)	5 (9.4)	16 (30.3)		
		Control	736	157	50	62.0±12.0	29.0±0.0		23 (27.7)	6 (7.2)	10 (12.0)	44 (53.0)		
Stefani [47]	2005	AD	396	397.5	66	72.2±8.1	18.2±1.7	493	Reference					2+
		Other	787	434	20	73.6±6.8	20.1±2.0		58 (67.4)	8 (9.3)	8 (9.3)	12 (14.0)		
Hampel [48]	2005	a-MCI	678	304	52	72.8±5.3	22.4±5.7	679	Reference					2+
		AD	545	230	93	72.5±8.3	28.9±1.0		24 (46.2)	10 (19.2)	5 (9.6)	13 (25.0)		
		Control	962	182	10	67.7±7.7	29.5±0.5		-	-	-	-		
Perneczky [49]	2011	a-MCI	622.95	275.61	21	67.9±8.8	27.7±0.0	-	Reference					2–
		na-MCI	789.91	38.12	35	61.9±7.7	27.5±0.0		17 (30.4)	7 (12.5)	4 (7.1)	28 (50.0)		
Lewczuk [50]	2007	a-MCI	172.6	53.5	106	67.7±8.2	-	-	Reference					2–
		na-MCI	228	37.35	49	59.7±8.5	-		63 (40.6)	18 (11.6)	43 (27.7)	31 (20.1)		

Abbreviations: a-MCI, amnestic mild cognitive impairment; na-MCI, non-amnestic mild cognitive impairment; AD, Alzheimer’s disease; non-AD, non-AD dementia; N, sample size; TP, True Positive; FP, False Positive; FN, False Negative; TN, True Negative.

*All biochemical measurements, *pg/ml*

### 1. Results of systematic literature review

**Fig 2:**
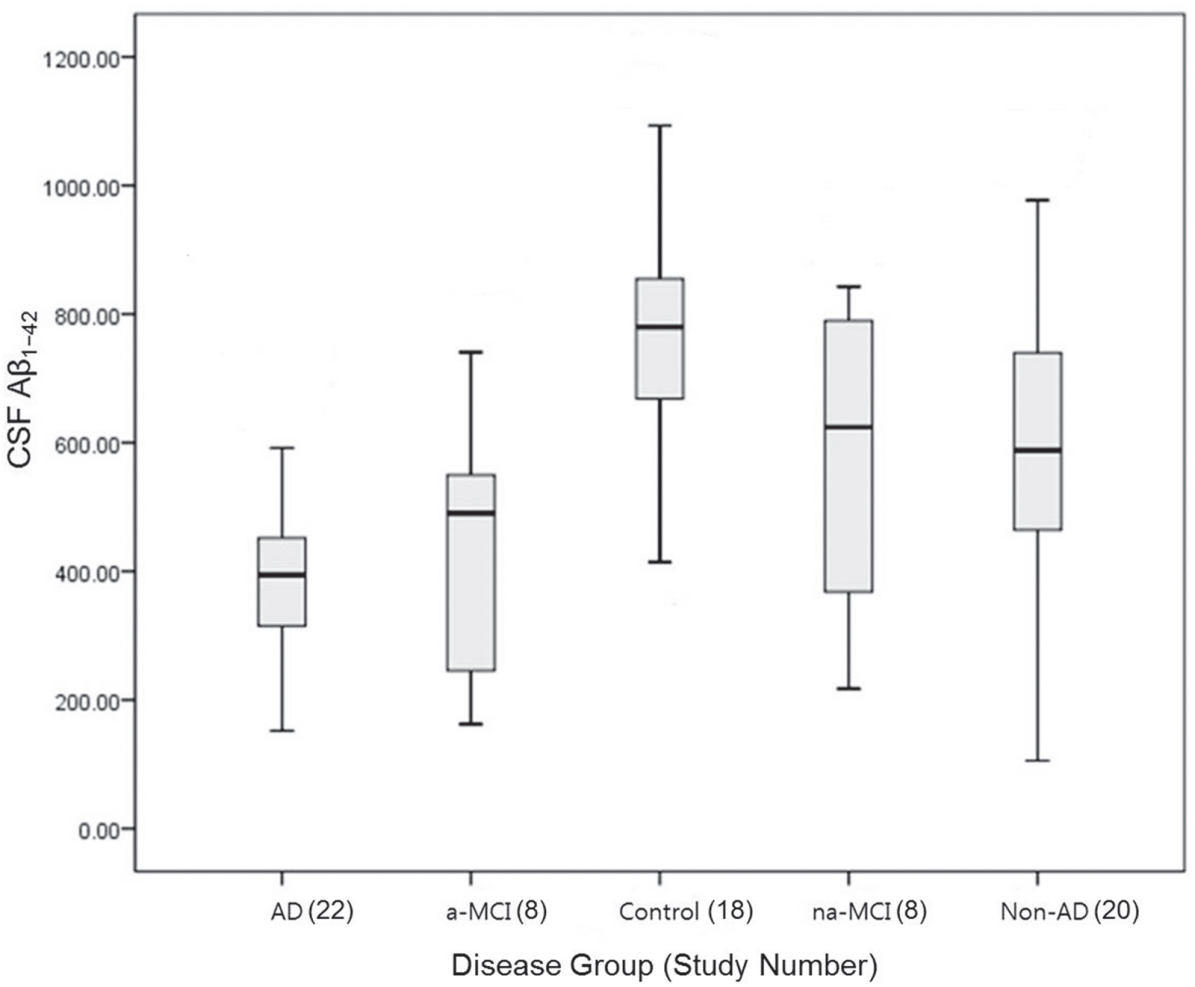
CSF Aβ_1–42_ levels with 95% confidence intervals. CSF Aβ_1–42_ levels in AD was 382.2 ± 102.0 pg/ml (95% CI 336.9–427.4) whereas levels in the healthy control group was 755.6 ± 209.1 pg/ml (95% CI 651.5–859.6). However, CSF Aβ_1–42_ levels in non-AD (589.0 ± 217.5, 95% CI 105.4–977.2 pg/ml), a-MCI (434.4 ± 200.6, 95% CI 162.4–740.8 pg/ml) and na-MCI (577.9 ± 244.6, 95% CI 217.5–842.5 pg/ml) frequently overlapped. Abbreviations: a-MCI, amnestic mild cognitive impairment; na-MCI, non-amnestic mild cognitive impairment; AD, Alzheimer’s disease; non-AD, non-AD dementia.

**Fig 3:**
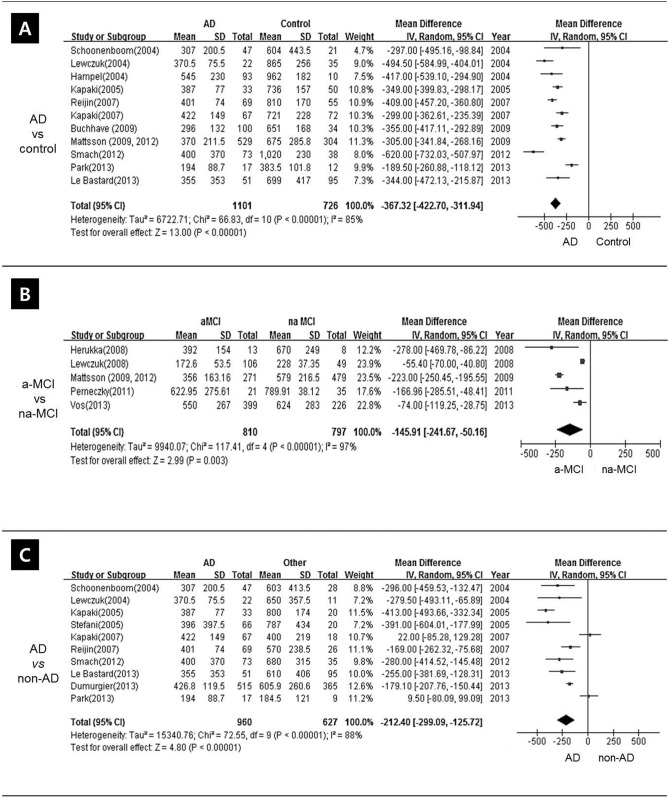
Forest plot of CSF Aβ_1–42_ levels. Pooled mean difference (MD) analysis of CSF Aβ_1–42_ levels revealed that overall levels were significantly lower in AD patients than in healthy controls. However, there was significant heterogeneity and the ranges frequently overlapped. Abbreviations: a-MCI, amnestic mild cognitive impairment; na-MCI, non-amnestic mild cognitive impairment; AD, Alzheimer’s disease; non-AD, non-AD dementia.

The diagnostic efficacy of CSF Aβ_1–42_ in AD and healthy controls was reported in eleven documents. CSF Aβ_1–42_ levels in AD ranged from 194±88.7 to 545±230 pg/ml, whereas levels in the healthy control group ranged from 383.5±101.8 to 1020±230 pg/ml (p <.001) (Figs. 2, 3 and Table 3). Five papers reported diagnostic efficacy of CSF Aβ_1–42_ for amnestic type MCI (a-MCI) patients and non-amnestic MCI (na-MCI). CSF Aβ_1–42_ levels ranged from 172.6±53.5 to 622.9±275.6 pg/ml in a-MCI, whereas levels in na-MCI ranged from 228±37.35 to 789.9±38.12 pg/ml (p = .003) (Figs. 2, 3 and Table 3). Diagnostic efficacy of CSF Aβ_1–42_ in non-AD dementias and AD was reported in 10 studies. CSF Aβ_1–42_ levels in AD ranged from 194±88.7 to 426.8±119.5pg/ml whereas levels in non-AD dementias ranged from 184.5±121 to 800±174 pg/ml (p <. 0001) (Figs. 2, 3 and Table 3). CSF Aβ_1–42_ level with 95% confidence intervals in AD was 382.2±102.0 pg/ml (95% CI 336.9–427.4) whereas levels in the healthy control group was 755.6±209.1 pg/ml (95% CI 651.5–859.6). However, CSF Aβ_1–42_ levels in non-AD (589.0 ± 217.5, 95% CI 105.4–977.2 pg/ml), a-MCI (434.4 ± 200.6, 95% CI 162.4–740.8 pg/ml) and na-MCI (577.9 ± 244.6, 95% CI 217.5–842.5 pg/ml) frequently overlapped (Figs. 2, 3 and Table 3).

### 2. Meta-analysis

**Fig 4:**
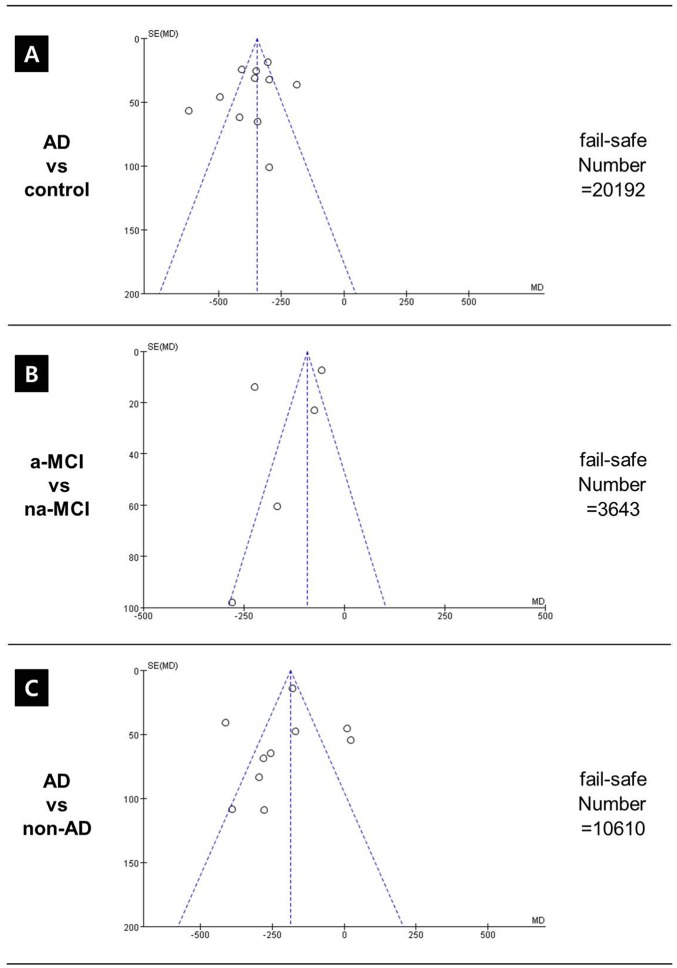
A funnel plot confirming heterogeneity of studies. There was significant heterogeneity between AD and healthy controls. Abbreviations: a-MCI, amnestic mild cognitive impairment; na-MCI, non-amnestic mild cognitive impairment; AD, Alzheimer’s disease; non-AD, non-AD dementia.

A funnel plot confirming heterogeneity of studies is presented in Fig. 4. Pooled mean difference (MD) analysis of CSF Aβ_1–42_ levels revealed that overall levels were significantly lower in AD patients than in healthy controls. However, there was significant heterogeneity and the ranges frequently overlapped: pooled MD was -367.32 (95%CI–422.70~–311.94), *p* < 0.001, I^2^ = 85%, effect Z = 13.00 (Fig. 3).

**Fig 5:**
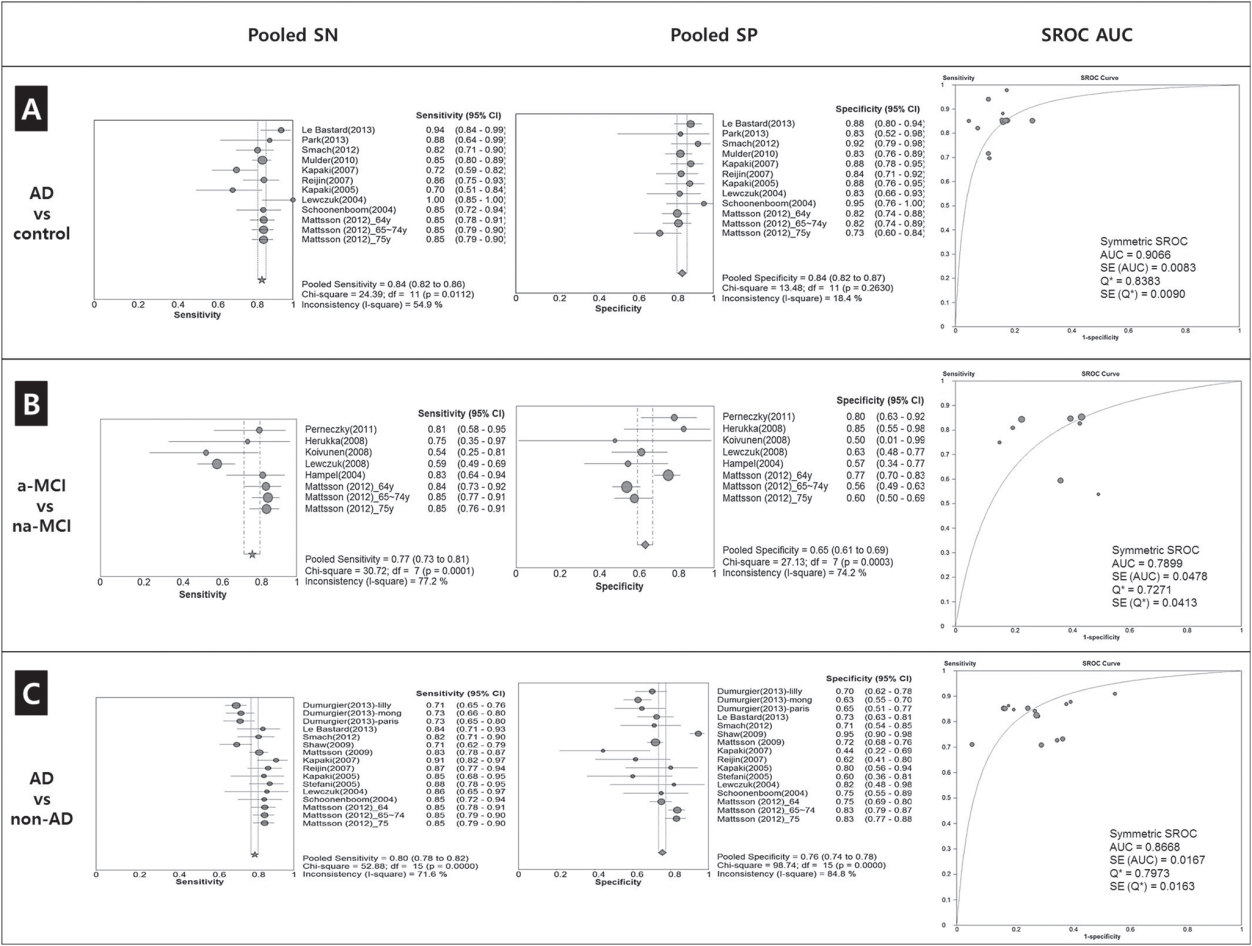
Forest plot of sensitivities and specificity and Receiver operating characteristics (ROC) curve for AD and control (A). Diagnostic accuracy was evaluated on the basis of ten documents: pooled sensitivity (SN) was 0.84 (95% CI 0.82–0.86), χ^2^ = 24.39, *p* = 0.0112, I^2^ = 54.9%, and pooled specificity (SP) was 0.84 (95% CI 0.82–0.87), χ^2^ = 13.48, *p* = 0.263, I^2^ = 18.4%. The SROC AUC (Summary Receiver Operating Characteristic Area Under the Curve (SROC AUC) value was 0.9066±0.0083. Forest plot of sensitivities and specificity and Receiver operating characteristics (ROC) curve for a-MCI and na-MCI (B). Diagnostic accuracy was evaluated on the basis of ten documents: pooled sensitivity (SN) was 0.77 (95% CI 0.73–0.81), χ^2^ = 30.72, *p* = 0.0001, I^2^ = 77.2%, and pooled specificity (SP) was 0.65 (95% CI 0.61–0.69), χ^2^ = 27.13, *p* = 0.003, I^2^ = 74.2%. The SROC AUC (Summary Receiver Operating Characteristic Area Under the Curve (SROC AUC) value was 0.7899±0.0478. Forest plot of sensitivities and specificity and Receiver operating characteristics (ROC) curve for AD and non-AD dementia (C). Diagnostic accuracy was evaluated on the basis of ten documents: pooled sensitivity (SN) was 0.80 (95% CI 0.78–0.82), χ^2^ = 52.88, *p* = 0.0000, I^2^ = 71.6%, and pooled specificity (SP) was 0.76 (95% CI 0.74–0.78), χ^2^ = 98.74, *p* = 0.0000, I^2^ = 84.8%. The SROC AUC (Summary Receiver Operating Characteristic Area Under the Curve (SROC AUC) value was 0.8668±0.0167. Abbreviations: AD, Alzheimer’s disease; non-AD, non-AD dementia; df, differences; SROC, Summary Receiver-Operating Characteristic curve; AUC, area under curve; SE, Standard Error; Q*, Heterogeneity statistic.

Diagnostic accuracy was evaluated on the basis of ten documents: pooled sensitivity (SN) was 0.84 (95% CI 0.82–0.86), χ^2^ = 24.39, *p* = 0.0112, I^2^ = 54.9%, and pooled specificity (SP) was 0.84 (95% CI 0.82–0.87), χ^2^ = 13.48, *p* = 0.2630, I^2^ = 18.4%. The SROC AUC (Summary Receiver Operating Characteristic Area Under the Curve (SROC AUC) value was 0.9066±0.0083 (Fig. 5).

Pooled MD analysis showed statistically significant higher CSF Aβ_1–42_ levels in na-MCI compared a-MCI groups, although highly heterogeneity was apparent: pooled MD was -145.91 (95%CI–241.67~–50.16), *p* = 0.003, I^2^ = 97%, effect Z = 2.99) (Fig. 3).

The diagnostic accuracy of CSF Aβ_1–42_ levels was evaluated on the basis of 8published reports. Pooled SN of CSF Aβ_1–42_ levels was 0.77 (95% CI 0.73–0.81), χ^2^ = 30.72, p = .0001, I^2^ = 77.2% and pooled SP was 0.65 (95% CI 0.61–0.69), χ^2^ = 27.13, p = 0.0003, I^2^ = 74.2%. The SROC AUC value was 0.7899±0.0478 (Fig. 5)

Pooled MD analysis demonstrated that CSF Aβ_1–42_ levels were significantly lower in patients with AD versus non-AD dementia, but the results were significantly heterogeneous: the pooled MD was -212.40 (95% CI -299.09~–125.72), *p*<.00001, I^2^ = 88%, effect Z = 4.80 (Fig. 3).

Diagnostic accuracy was evaluated based on 16 reports. Pooled SN was 0.80 (95% CI 0.78–0.82), χ^2^ = 52.88 (p = .0000), I^2^ = 71.6%, and pooled SP was 0.76 (95% CI 0.74–0.78), χ^2^ = 98.74 (p = .0000), I^2^ = 84.8%. The SROC AUC value was 0.8668±0.0167 (Fig. 5).

Additionally, a sub-analysis according to age and MMSE was performed to determine the cause of the heterogeneity within the effect size of the difference between AD and non-AD. There were no significant findings (S1 Fig.).

The diagnostic accuracy of CSF Aβ_1–42_ levels in a-MCI versus AD, and a-MCI versus healthy controls, was only reported in one document and meta-analysis could therefore not be performed.

## Discussion

In this study we have evaluated the clinical utility of CSF Aβ_1–42_ levels in the diagnosis of AD versus healthy controls and non-AD dementias. Data retrieved from systematic literature review did not identify threshold CSF Aβ_1–42_ levels that can distinguish between healthy controls and subjects with AD because there was highly significant heterogeneity and the ranges frequently overlapped. The fact that there is not a threshold, in other words a cut off, which can distinguish AD from healthy controls, as well from the other categories analyzed should be highlighted and it is a result of the meta-analysis along with those reported. However, this meta-analysis confirms that, overall, CSF Aβ_1–42_ levels in AD are significantly lower than in healthy controls.

Although meta-analysis was unable to differentiate reliably between a-MCI and healthy controls, several reports have attested to the clinical utility of CSF Aβ_1–42_ levels in MCI. Maruyama *et al*. reported that CSF Aβ_1–42_ levels did not differ significantly between the healthy control group and MCI [17]. Another study showed the values of CSF Aβ_1–42_ were significantly lower in the progressive MCI group than in the control subjects and the stable MCI group [18]. CSF Aβ_1–42_ concentration has a high diagnostic accuracy for correct allocation of AD patients in case—control studies and, together with CSF tau levels, can predict incipient AD in patients with MCI [19]. Values of CSF Aβ_1–42_ differed according to sample state (fresh versus frozen samples), but overall values were lower in AD patients than in MCI patients [20]. However, a threshold value discriminating between a-MCI and healthy controls could not be established. Instead, other studies have employed the ratio of CSF Aβ_1–42_ to either Aβ_1–40,_ total tau, or phosphorylated tau as a potential measure of the evolution of MCI to AD [19,21–24].

In the present analysis there were significant differences between the a-MCI and na-MCI groups. CSF Aβ_1–42_ levels were lower in a-MCI (range 172.6±53.5 to 622.9±275.6 pg/ml) than in na-MCI (range 228.0±37.35 to 789.9±38.12pg/ml), and the pooled MD between groups was significant (pooled MD, 59.77 pg/ml). However, there was highly significant heterogeneity (I^2^ = 66%), and calculated diagnostic accuracy for MCI alone gave SN and SP values, respectively, of 0.52–0.83 and 0.50–0.84.

Significant discriminatory power was also seen in AD versus non-AD dementia. CSF Aβ_1–42_ levels in AD (range 194.0±88.7 to 545.0±230.0 pg/ml) were significantly below those reported in non-AD dementia (range 184.5±121.0 to 800.0 ± 174.0 pg/ml). The pooled MD value between groups was significantly lower in AD (pooled MD, 187.21 pg/ml). However, there was also significant heterogeneity (I^2^ = 66%), and the calculated diagnostic accuracy of AD versus non-AD dementia gave SN and SP values, respectively, of 0.71–0.91 and 0.44–0.82.

These findings may be summarized as follows. First, in patients with probable AD, CSF Aβ_1–42_ levels are of value in differential diagnosis of AD from other dementias and from healthy controls. The mean concentration of Aβ_1_–_42_ in the CSF is significantly reduced by around 50%, in subjects with AD relative to age-matched controls [4,25]. There are debates about whether the Aβ_1–42_ alone is useful or not in differentiating AD from non-AD dementias including frontotemporal dementia, vascular dementia, and dementia with Lewy bodies (DLB). Because concurrent presence of fibrillar Aβ deposits occurs in the majority of patients with DLB, it is possible that the reduced Aβ_1_–_42_ levels in the CSF have also been documented in patients with other dementia [4]. However, meta-analytic study indicates that CSF Aβ_1–42_ can serve as a diagnostic and surrogate biomarker for Aβ deposition in the brain [26]. Second, the ranges of Aβ_1–42_ levels partially overlap between AD and a-MCI, and it is therefore not possible to establish a cut-off value that discriminates between the two groups. Moreover, it is possible that a-MCI is an extension of AD pathology, and it has been suggested that a-MCI might be redefined to as a-MCI due to AD [6]. There might be the following several reasons; Some outstanding prospective CSF studies in MCI subjects would be particularly useful to add strength to this claim [27,28]. However, we decided to enroll papers published since 2004, because the criteria for MCI were revised to encompass other patterns of cognitive impairment in addition to memory loss [29]. In this paper we analyzed CSF results according to a-MCI and na-MCI. The other is considerable intra- or inter-laboratory variability of CSF analyses, which may influence the diagnostic classification of dementia according to results of CSF [30]. The intra- and inter-laboratory variability in CSF results from differences in pre-analytical and analytical procedures, lot-to-lot variation of analytical kits, freezing conditions and storage time [31–33]. It is necessary for research community to overcome this confusing situation that CSF variability was largest for Aβ_1–42_.

In summary, this meta-analysis establishes that reduced Aβ_1–42_ levels are of diagnostic utility in AD, and relatively high CSF levels of Aβ_1–42_ are indicative of non-AD pathology (e.g., na-MCI, non-AD dementias). However, CSF Aβ_1–42_ levels alone are insufficient for reliable differential diagnosis of AD. Further research on the use of combinations of biomarkers, for example Aβ_1–42_ levels in conjunction with other markers (e.g., total Aβ, Aβ_1–40_, tau, phosphorylated tau), will be necessary in order to develop CSF biochemical measurements permitting reliable diagnosis of AD versus other non-AD cognitive impairments.

## Supporting Information

S1 Fig Sub-group analysis by age and MMSE in the groups of AD and non-AD. A sub-analysis according to age and MMSE has performed to determine the cause of the heterogeneity within the effect size of the difference between AD and non-AD. There were no significant findings. Abbreviations: AD, Alzheimer’s disease; non-AD, non-AD dementia.
(TIF)

S1 PRISMA Checklist For meta-analyses and systematic reviews, a PRISMA checklist. (DOC)

S1 Table Ovid-MEDLINE and EMBASE Search Strategy. Literature searches using MEDLINE and EMBASE. Abbreviation: PICO, Patients—Intervention—Comparators—Outcomes.
(DOCX)
