# Supplementary figures and images for: Cerebrospinal Fluid β-Amyloid1–42 Levels in the Differential Diagnosis of Alzheimer’s Disease—Systematic Review and Meta-Analysis
Source: PLoS One. 2015 Feb 24;10(2):e0116802. doi: 10.1371/journal.pone.0116802 (PMC4339391; doi:10.1371/journal.pone.0116802)

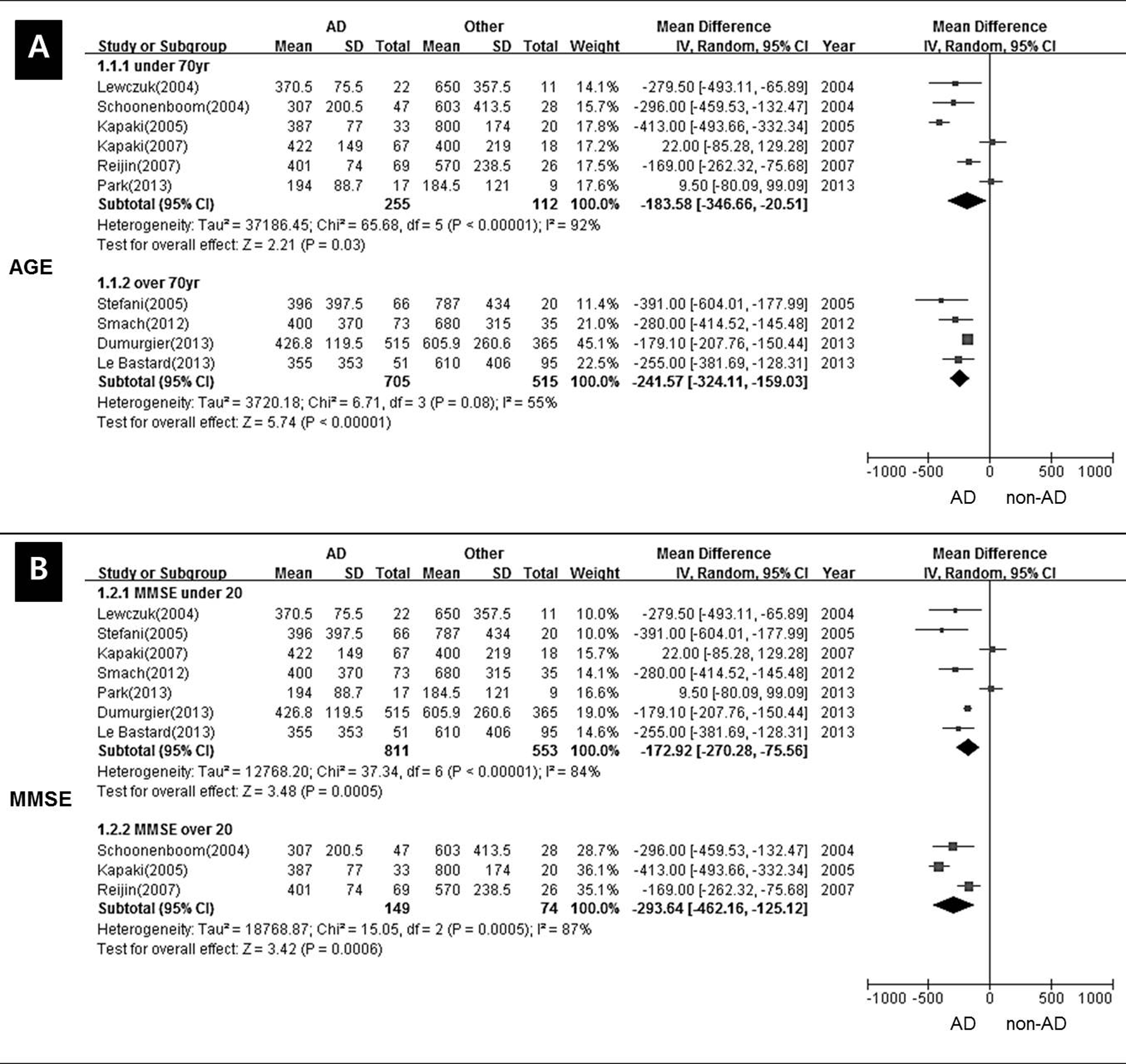

Supplement: S1 Fig — A sub-analysis according to age and MMSE has performed to determine the cause of the heterogeneity within the effect size of the difference between AD and non-AD. There were no significant findings. Abbreviations: AD, Alzheimer’s disease; non-AD, non-AD dementia. (TIF) [file pone.0116802.s001.tif]
