# Supplementary material for: Cerebrospinal Fluid β-Amyloid1–42 Levels in the Differential Diagnosis of Alzheimer’s Disease—Systematic Review and Meta-Analysis
Source: PLoS One. 2015 Feb 24;10(2):e0116802. doi: 10.1371/journal.pone.0116802 (PMC4339391; doi:10.1371/journal.pone.0116802)
Supplement: S1 Table — Literature searches using MEDLINE and EMBASE. Abbreviation: PICO, Patients—Intervention—Comparators—Outcomes. (DOCX) [file pone.0116802.s003.docx]

**Supplementary Table 1 Ovid-MEDLINE and EMBASE Search Strategy**

| **PICO** | **No** | **Search term** | **Searched literature (*n*)** | |
| --- | --- | --- | --- | --- |
|  |  |  | **MEDLINE** | **EMBASE** |
| Patients | 1 | dementia.mp. or dementia/ | 84,165 | 115,778 |
|  | 2 | Alzheimer disease/ or alzheimer.mp. | 73,731 | 123,948 |
|  | 3 | cognitive impairment.mp. or mild cognitive impairment/ | 25,886 | 39,463 |
|  | 4 | 1 OR 2 OR 3 | 147,525 | 217,553 |
| Index test | 5 | ((beta or amyloid) adj2 42).mp. | 97 | 103 |
|  | 6 | (amyloid adj2 (beta or 42)).mp. | 474 | 623 |
|  | 7 | 10 OR 11 | 24,805 | 26,745 |
|  | 8 | cerebrospinal fluid.mp. or cerebrospinal fluid/ | 75,623 | 135,751 |
|  | 9 | CSF.mp. or cerebrospinal fluid/ | 85,509 | 138,707 |
|  | 10 | ((Lumbar or spinal) and puncture).mp. | 9,491 | 17,424 |
|  | 11 | 8 OR 9 OR 10 | 128,395 | 189,995 |
|  | 12 | 7 AND 11 | 2,716 | 4,773 |
| P+I | 13 | 4 and 12 | 2,081 | 3,982 |
|  | 14 | ANIMALS/ | 5,486,090 | 1,890,932 |
|  | 15 | HUMANS/ | 13,631,608 | 14,862,188 |
|  | 16 | 14 AND 15 | 1,528,202 | 482,645 |
|  | 17 | 15 NOT 16 | 3,957,888 | 1,408,287 |
|  | 18 | 13 NOT 17 | 2,006 | 3,972 |
|  | 19 | elisa.mp. or enzyme-linked immunosorbent assay/ | 181,268 | 238,511 |
|  | 20 | immunoassay.mp. or immunoassay/ | 56,681 | 101,986 |
|  | 21 | Pib AND (PET or positron emission tomography.mp.) | 477 | 1,023 |
|  | 22 | (biopsy or autopsy).mp. | 393,696 | 693,753 |
|  | 23 | Innotest OR Biosource OR AlzBio | 120 | 575 |
|  | 24 | 19 OR 20 OR 21 OR 22 OR 23 | 800,265 | 1,206,161 |
|  | 25 | 19 AND 24 | 496 | 957 |
| **TOTAL** |  |  | **496** | **957** |
|  | | | | |
